# A comparative profitability analysis of transcatheter versus surgical aortic valve replacement in a high-volume French hospital

**DOI:** 10.1186/s13561-019-0223-0

**Published:** 2019-02-14

**Authors:** François Huchet, Jacques Chan-Peng, Fanny d’Acremont, Patrice Guerin, Gael Grimandi, Jean-Christian Roussel, Julien Plessis, Vincent Letocart, Thomas Senage, Thibaut Manigold

**Affiliations:** 1 Service de Cardiologie, Hôpital Nord Laennec, Unité d’Hémodynamique et Cardiologie Interventionnelle, CHU de Nantes, Boulevard Professeur Jacques Monod, 44800 Saint-Herblain, France; 2 Pharmacie Centrale, Hôpital Saint-Jacques, CHU de Nantes, 44093 Nantes, France; 3 Service de chirurgie cardio-thoracique, Hôpital Nord Laennec, CHU de Nantes, 44800 Saint-Herblain, France

**Keywords:** Economical/cost-effectiveness, Aortic valve disease, percutaneous intervention, Cardiovascular diseases

## Abstract

**Background:**

Current scientific guidelines have extended the indication for transcatheter aortic valve replacement (TAVR) to patients who present an intermediate risk for surgery and have been so far considered for conventional surgery. We previously demonstrated that the TAVR procedure generated profits despite elevated costs, but comparison with surgery has not been performed. The objective of this study was to assess the profitability of the TAVR procedure compared with conventional surgery in a high-volume French hospital.

Consecutive patients eligible for transfemoral TAVR or surgical aortic valve replacement (SAVR) were included retrospectively in this single-centre study between September 2014 and December 2015. The primary endpoint was the profitability of each procedure (defined as the ratio between the profit and total revenues), calculated for each patient. Secondary composite endpoints included major adverse events in the 30 days following procedure and breakdown of costs.

**Results:**

Two hundred and thirty-eight patients were included in the TAVR group and 341 in the SAVR group. TAVR patients presented higher operative risk scores and more comorbidities. Compared with SAVR, TAVR was associated with higher profits (€2732 ± 1768 per patient vs. €2177 ± 2437 per patient, *P* < 0.001) but also higher costs (€27,778 ± 4961 vs. €17,813 ± 6071, P < 0.001) resulting in lower profitability (9.3 ± 5.7% vs. 11.7 ± 10.1%, *P* < 0.001). The price of the bioprosthesis represented 70% of the TAVR total cost.

**Conclusions:**

TAVR performed in carefully selected patients was associated with higher profits than SAVR, but also higher costs resulting in lower profitability.

## Introduction

Transcatheter aortic valve replacement (TAVR) has revolutionised the prognosis of patients who present with severe aortic valve stenosis and cannot undergo conventional surgery. The non-inferiority of TAVR versus surgical aortic valve replacement (SAVR) has been demonstrated in high-risk patients [1], and recent studies are widening its use to patients with high-to-intermediate risk [2]. Current guidelines [3] support its use in symptomatic patients who are considered unsuitable for surgery as well as in intermediate risk patients with a favourable transfemoral access.

Consequently, the number of TAVR procedures is rapidly increasing. In 2016, in our centre, the implantation of percutaneous aortic bioprostheses became almost as frequent as the implantation of conventional isolated surgical bioprostheses, leading to rising costs and hospitalisations. We previously reported the elevated costs of the TAVR procedure [4], which are associated with large benefits for the institution. However, no direct comparison of cost-revenue has been performed between TAVR and conventional SAVR. TAVR prostheses are very expensive, and their implantation as a first-choice strategy in low-to-intermediate risk patients may be questionable in an era when healthcare costs are becoming a major concern. In this context, a comparative analysis could support the choice of the ideal strategy in patients eligible for both TAVR and SAVR.

Therefore, the objective of this study was to perform a comparative cost-revenue analysis of the TAVR and SAVR procedures in real-world patients managed in a high-volume French hospital.

## Methods

### Study population

Eligible patients were those scheduled to undergo transfemoral TAVR or SAVR using bioprostheses at the University Hospital of Nantes, France. The therapeutic choice was performed after an exhaustive preoperative appraisal by the heart team, in accordance with the European Society of Cardiology guidelines [5]. All consecutive eligible patients identified between September 2014 and December 2015 were included retrospectively into the study. Clinical data were collected in our local database. Data from TAVR patients were exported to the FRANCE-TAVI registry (managed by the French Society of Cardiology). All patients gave consent to the use of their data.

### Interventions and early follow-up

Transfemoral TAVR procedures was performed using the balloon-expandable SAPIEN 3® valve (Edwards Lifesciences, Irvine, CA, USA) or the self-expendable COREVALVE EVOLUT-R® valve (Medtronic, Minneapolis, Minnesota, USA). The procedures were realised by two senior interventional cardiologists, as described previously [6]. The implantation of the percutaneous valves was systematically preceded by balloon dilatation of the native aortic valve. No pre-dilatation was performed in valve-in-valve procedures. A single dose of heparin (0.5 mg/kg) was injected immediately after positioning the major transarterial access, with no control of activated clotting time.

SAVR procedures were performed by a senior heart surgeon, with the assistance of a resident. The operating techniques and use of cardioplaegia were decided by the operating surgeon, but a surgical approach via full median sternotomy was systematically used. After surgery, patients were carefully monitored in intensive care unit (ICU) for a few hours, and then in a conventional unit prior to hospital discharge.

The antithrombotic regimen was delivered in accordance with concurrent guidelines [5]. Echocardiography was performed regularly during hospitalisation and before hospital discharge.

### Cost-revenue analysis

Medicoeconomic data were collected from the French National Health Society and from the Medical Programme of Information Systems (PMSI: Programme de médicalisation des systèmes d’information).

For each patient, we calculated the total cost of the procedure (TAVR or SAVR), and compared it with the reimbursement received by the hospital. The costs were assessed using the 2014 French National Scale of Health Costs (available online http://www.scansante.fr/applications/enc-mco) and were dependent on length of hospital stay, development of early complications and requirement for a permanent pacemaker after the TAVR.

Four different costs were added to provide a total cost per patient: the clinical cost, which included all costs related to the hospitalisation unit (ICU and/or standard unit); the medicotechnical cost, which included all costs related to the invasive procedure, pacemaker implantation (if performed) and standard laboratory and imaging tests (this cost also included the remuneration of doctors and nurses during the procedure, and the maintenance and depreciation costs of the equipment); the logistical cost, which included medical (pharmacy, hygiene) and general (laundry, nourishment, administration, patient handling) logistical costs; and the direct cost, which included the price of the device(s), medicines and consumables.

The cost of medical devices and blood products and the remuneration of doctors and nurses during the procedure were estimated for each patient using a microcosting analysis. The total revenue for the hospital were determined for each patient as the sum of three separate revenues: a standard reimbursement defined by the patient’s severity level; a supplement for patients monitored in an ICU or reanimation unit; and a reimbursement of implanted devices (in the TAVR group).

### Endpoints

The primary endpoint was the profitability associated with the TAVR and SAVR procedures. We estimated the profit by calculating the difference between costs and revenues associated with each patient. Profitability was then calculated as the ratio between profit and total revenues for each patient.

The economic secondary endpoint was the breakdown of costs (clinical, medicotechnical, logistical and direct costs) for each treatment option.

The clinical secondary endpoints were major adverse events within 30 days of the procedure, defined according to the Valve Academic Research Consortium (VARC)-2 criteria [7], and including major vascular complications, major/life-threatening bleeding, major adverse postoperative events (defined as stroke and/or myocardial infarction), early rehospitalisation for heart failure, pacemaker requirement, tamponade and stage 3 acute kidney injury, analysed as separate endpoints.

### Subgroup analysis

In the TAVR group, two subgroups were considered depending on the prostheses being implanted: SAPIEN 3 vs. COREVALVE. In the SAVR group, two subgroups were determined based on need for invasive cardiac monitoring (ICM).

### Statistical analysis

Continuous variables are reported using means ± standard deviations and were compared using Student’s *t* test. We used the χ^2^ test or Fisher’s exact test to compare categorical variables. A two-sided *P* value < 0.05 was considered to indicate statistical significance. Statistical analyses were performed with SPSS® software, version 20.0 (IBM Corp., Armonk, NY, USA).

## Results

### Study population

Between September 2014 and December 2015, the heart team prescribed transfemoral TAVR in 238 patients and SAVR 341 patients. The procedure was urgent in 15 TAVR patients (6.3%) and in 14 SAVR patients (4.1%; *P* = 0.23).

### Baseline characteristics

Demographic and preoperative characteristics are presented in Table 1. Patients undergoing TAVR were significantly older than those selected for SAVR (81.8 ± 7.2 years vs. 74.5 ± 7.5 years; *P* < 0.001). Fewer men were present in the TAVR group (49% vs 59%, *P* = 0.02). Comorbidities were more frequent, and symptoms were more severe in the TAVR group, resulting in significantly higher operative risk scores.

**Table 1:** Demographic and preoperative characteristics

	SAVR (*N* = 341)	TAVR (*N* = 238)	*P*-values
Male sex	201 (59%)	118 (49%)	0.02
Age, years	74.5 ± 7.5	81.8 ± 7.2	< 0.001
NYHA functional class I/II/III/IV	57/217/57/10	10/129/73/26	< 0.001
Logistic Euroscore, %	6.9 ± 4.9	14.7 ± 9.4	< 0.001
Euroscore II, %	2.3 ± 2.3	4.8 ± 3.6	< 0.001
Supraventricular arrhythmia	32 (9%)	89 (37%)	< 0.001
Coronary artery disease	76 (22%)	85 (36%)	< 0.001
Peripheral vascular disease	24 (7%)	50 (21%)	< 0.001
Chronic obstructive pulmonary disease	16 (5%)	54 (23%)	< 0.001
Permanent pacemaker	4 (1%)	21 (9%)	< 0.001
Chronic kidney disease	18 (5%)	116 (49%)	< 0.001
Liver disease	1 (0.3%)	12 (5%)	< 0.001
History of cancer	11 (3%)	40 (17%)	< 0.001
LVEF, %	61.8 ± 9.1	56.3 ± 12.3	< 0.001
Mean transaortic gradient in aortic stenosis, mmHg	55.2 ± 13.0	51.0 ± 19.3	0.005

Values are expressed as number of patients (percentage) or mean ± standard deviation

*LVEF* left ventricular ejection fraction, *NYHA* New York Heart Association, *SAVR* surgical aortic valve replacement, *TAVR* transcatheter aortic valve replacement

### Procedure outcomes and length of stay

In the TAVR group, 189 patients received a SAPIEN 3 prosthesis and 49 patients received a COREVALVE prosthesis. No switch to surgical procedure was necessary and a large majority of patients were operated under local anaesthesia (214/238, 90%). In the SAVR group, 101 patients out of 341 required ICM. Procedural success was high in both groups (237/238 in the TAVR group vs. 341/341 in the SAVR group). One severe aortic regurgitation was observed in the TAVR group (none in the SAVR group). Three patients (1.2%) died within 30 days in the TAVR group vs. four (1.2%) in the SAVR group (*P* = 0.99). The cause of death in the TAVR group was cardiac for one patient (annulus rupture during TAVR) and non-cardiac for two patients (both occurring after hospital discharge). The cause of death in the SAVR group was cardiac for three patients. No significant difference was observed between groups regarding the length of stay in ICU, but the total length of stay in hospital was shorter in the TAVR group (9.0 ± 6.0 days vs. 11.7 ± 5.5 days; *P* < 0.001). No significant difference was observed regarding the main echocardiographic results following the procedure.

### Primary endpoint: Profitability analysis

**Fig. 1:**
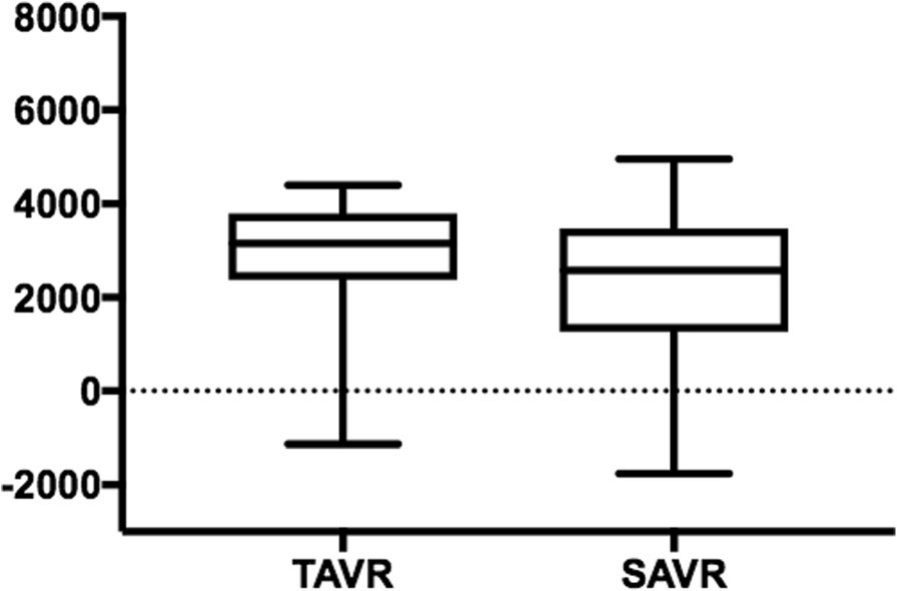
Profit analysis, € (median, quartiles, minimum and maximum) *P* < 0.001. SAVR: surgical aortic valve replacement, TAVR: transcatheter aortic valve replacement

**Fig. 2:**
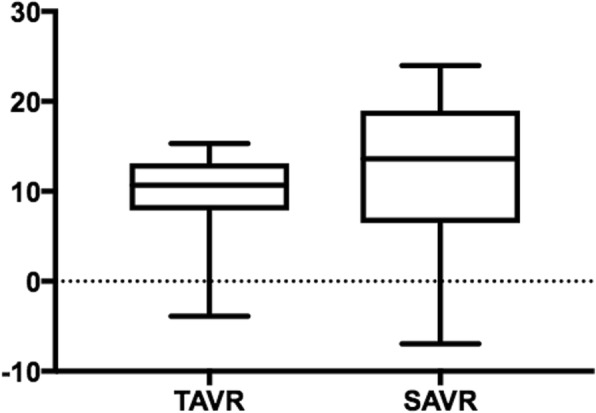
Profitability of the procedure, % (median, quartiles, minimum and maximum) *P <* 0.001. SAVR: surgical aortic valve replacement, TAVR: transcatheter aortic valve replacement

The profits associated with the TAVR and SAVR procedures are presented in Fig. 1. The profit observed for the TAVR group was significantly higher than for the SAVR group (€2732 ± 1768 vs €2177 ± 2437, *P* = 0.002). The mean costs per patient for the TAVR and SAVR procedures were €27,778 ± 4961 and €17,813 ± 6071, and the mean revenues per patient were €30,509 ± 3760 and €19,989 ± 4739 (all *P* < 0.001), respectively. Thus, the TAVR procedure presented an additional cost of €9965 (56%) per patient compared with the SAVR procedure, resulting in a reduced profitability (9.3 ± 5.7% vs. 11.7 ± 10.1%, *P* < 0.001, Fig. 2).

### Secondary economic endpoint: Breakdown of costs

**Fig. 3:**
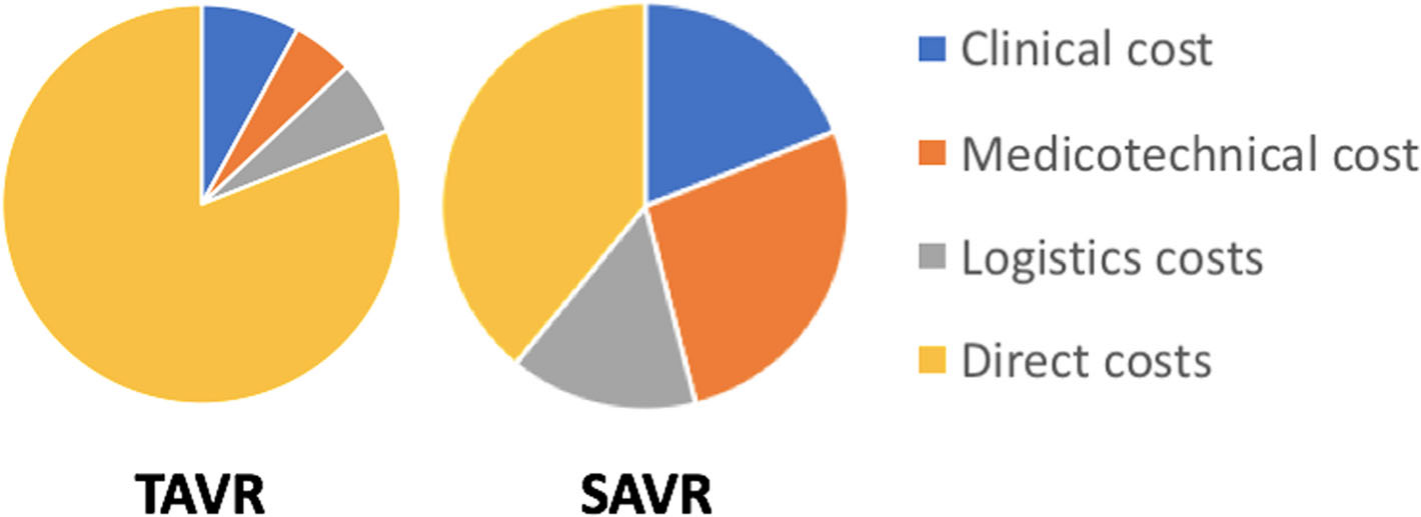
Overall breakdown of costs. SAVR: surgical aortic valve replacement, TAVR: transcatheter aortic valve replacement

The breakdown of costs is presented in Fig. 3. Significant differences were observed between the TAVR and SAVR groups regarding all considered costs (all *p* < 0.02). All costs were significantly lower for TAVR patients, except for direct costs, which were mostly driven by the price of the bioprosthesis representing 70% of the total cost and 87% of device-related costs. Logistical and medicotechnical costs contributed less than 12% to the total costs in the TAVR group, whereas they represented almost 40% in the SAVR group.

### Secondary clinical endpoint: Major adverse events

Major adverse events within 30 days of the procedure are presented in Table 2. Patients in the TAVR group required more frequently a permanent pacemaker implantation (17% vs. 3%, *P* < 0.001), and presented higher rates of disabling stroke or major vascular complications. SAVR patients required more frequently blood transfusions and prolonged mechanical ventilation.

**Table 2:** Major adverse events within 30 days of the procedure

	SAVR (*N* = 341)	TAVR (*N* = 238)	*P*-values
Disabling stroke	0 (0%)	4 (2%)	0.03
Myocardial infarction	2 (0.6%)	2 (0.8%)	0.70
Major vascular complications	0 (0%)	12 (5%)	< 0.001
Blood transfusion requirement	43 (13%)	15 (6%)	0.02
Respiratory failure requiring prolonged ventilation	30 (9%)	2 (0.8%)	< 0.001
Pacemaker requirement	9 (3%)	40 (17%)	< 0.001
Stage 3 acute kidney injury	4 (1%)	4 (2%)	0.72
Dialysis requirement	4 (1%)	1 (0.4%)	0.65
Early rehospitalization for heart failure	2 (0.6%)	6 (3%)	0.07

Values are expressed as number of patients (percentage)

*SAVR* surgical aortic valve replacement, *TAVR* transcatheter aortic valve replacement

### Subgroup analysis

In the TAVR group, the patients receiving a SAPIEN 3 prosthesis (*n* = 189) and those receiving a COREVALVE prosthesis (*n* = 49) had similar baseline characteristics (not shown), except for a more frequent prior aortic bioprosthesis implantation in the COREVALVE subgroup, due to our team preferring the implantation of a self-expandable prosthesis for the valve-in-valve procedures. No significant difference was observed between subgroups regarding the primary endpoint (profitability 9.4 ± 5.9% in the SAPIEN 3 patients vs. 8.8 ± 4.8% in the COREVALVE patients, *P* = 0.45) and the occurrence of major adverse events. The analysis of the breakdown of costs demonstrated significantly higher direct costs in the COREVALVE subgroup (€23,351 ± 5887 vs. €22,189 ± 1617; *P* = 0.02), caused by significantly higher device-related costs (€21,975 ± 5653 vs. €20,881 ± 1518; *P* = 0.02). No significant difference was observed regarding the requirement for a permanent pacemaker in the patients not implanted previously to the valvular procedure (33 patients out of 170 in the SAPIEN 3 subgroup vs. 7 out of 30 in the COREVALVE subgroup, *P* = 0.49).

In the SAVR group, the patients who required ICM following the surgery (*n* = 101) presented more frequent comorbidities. They also had a more severe New-York Heart Association (NYHA) functional class and a significantly longer hospital stay. The profitability associated with the procedure was significantly higher in the subgroup of patients who required ICM (14.9 ± 10.2% vs. 11.2 ± 8.7, *P* < 0.001), in line with higher hospital revenues (€22,879 ± 4779 vs €18,773 ± 3401, *P* < 0.001) and despite significantly higher costs (€19,732 ± 6275 vs. €17,005 ± 4528, *P* < 0.001),

## Discussion

Our study demonstrates that the TAVR procedure performed with the new generation of percutaneous prostheses is associated with higher profits for the hospital than conventional surgery, but it also involves significantly higher costs, which results in lower profitability. Our cohort included real-world patients recruited in a high-volume French centre and the results describe the current relative economic status of both procedures in the context of the French healthcare system.

The study population was representative of the patients seen in current practice. In the TAVR group, despite old age and comorbidities, most patients presented intermediate operative risk scores, as a result of careful and multidisciplinary selection [8, 9], whereas in the SAVR group, a large majority of patients had low operative risk scores. In line with recent studies and registries [10, 11], procedural success was high in both groups (237/238 in the TAVR group vs. 341/341 in the SAVR group). Seven patients (1.2%) died within 1 month of the procedure.

The percutaneous implantation of an aortic bioprosthesis has been associated with a reduction in hospital length of stay compared with conventional surgery, especially in elderly patients with comorbidities and patients who have suitable transfemoral access [12]. This observation was confirmed in our study, where TAVR patients had a significantly shorter hospital stay despite old age and numerous comorbidities. Some studies have considered the possibility of discharging patients within a few days of a TAVR procedure [13, 14]. This may minimally increase the profit generated from the procedure as the clinical costs only accounted for 9% of the total TAVR costs. On the other hand, the length of hospital stay following conventional surgery would be more complicated to shorten, and will continue to contribute significantly to the clinical costs, which account for 20% of the total SAVR costs.

Medicoeconomic data on TAVR and comparisons with SAVR are limited because of the broad diversity among national healthcare systems. Mitigated results regarding the balance between health costs and quality of life measured as quality-adjusted life years (QALY) have been published on the populations enrolled in the PARTNER studies from the US [12] and Canadian [15] perspectives. Transfemoral TAVR was found more cost-effective than conventional surgery, but the observed difference was marginal. Moreover, Tam et al. [15] reported that the small improvement in QALY observed with TAVR was counterbalanced by an increase in total lifetime costs estimated to around CAD 10,000 per patient. In this context of slight benefits associated with important increases in costs, it seems difficult to broaden the TAVR procedure to low risk patients.

To our point of view, as long as the TAVR remain that expensive, the specific associated issues should be solved before enlarging the indication. These include uncertainty on bioprosthesis durability and choice of the optimal antithrombotic regimen (especially in the context of concern regarding prostheses thromboses) as well as the frequent requirement for permanent pacemaker implantation. On the other hand, a large part of the TAVR overall cost is attributed to the price of the bioprosthesis and the implantation of a pacemaker further increases the procedural cost. We believe a reduction in the total cost of the TAVR procedure would mostly be achieved by significantly reducing the price of the device and limiting the need for pacemaker implantation.

The subgroup analysis comparing procedural costs with the SAPIEN 3 and COREVALVE prostheses demonstrated significantly higher direct costs and a trend for higher total costs with the COREVALVE prosthesis. The COREVALVE prosthesis implantation has been associated with a moderate increased risk of pacemaker implantation compared with balloon-expandable valve replacement [16]. Yet, in our small subgroup analysis, pacemaker requirement rates were not significantly different between TAVR subgroups and could not explain the increase in device-related costs observed in the COREVALVE subgroup. The increase in device-related costs were probably related to the requirement of a separate introducer, a separate balloon for the predilatation of the native aortic valve, and more frequent necessity of post-dilatation in the self-expendable subgroup.

In the surgical group, the requirement for ICM was associated with significantly higher profits, which further emphasises the low profit to be derived from conventional surgery in the absence of ICM. Moreover, patients requiring ICM are likely to have higher operative risk and become candidates for a future TAVR procedure.

### Study limitations

We produced a single-centre study, but this limitation was balanced by high volume and use of standard patient recruitment. The length of hospital stay was long in both groups during the study time, contributing to limit the profitability of both techniques. Regardless of these limitations, both techniques were found to be highly profitable for the hospital. Finally, the price of the TAVR devices recently decreased to €14,283 (application date: August 2018). This will reduce the cost of the TAVR procedure that still remains more expensive than the SAVR procedure but could demonstrate higher profitability in a near future.

## Conclusion

This study demonstrates that TAVR generates slightly higher profits than SAVR in an academic high-volume hospital. However, total costs are significantly increased for a TAVR procedure resulting in lower profitability. A large part of the TAVR cost is related to the price of the bioprosthesis. If the indication for TAVR is to be extended to patients with low operative risk, technical issues such as pacemaker requirement will have to be addressed and the price of the device will have to decrease.

## Abbreviations

ICM: Invasive cardiac monitoring
ICU: Intensive care unit
PMSI: Medical Programme of Information Systems (Programme de médicalisation des systèmes d’information)
QALY: Quality-adjusted life years
SAVR: Surgical aortic valve replacement
TAVR: Transcatheter aortic valve replacement
